# Supplementary material for: Spatiotemporal dissection of the Golgi apparatus and the ER-Golgi intermediate compartment in budding yeast
Source: eLife. 2024 Mar 19;13:e92900. doi: 10.7554/eLife.92900 (PMC10950332; doi:10.7554/eLife.92900)
Supplement: Supplementary file 1. [file elife-92900-supp1.pdf]

```
Consensus       ---------------XXGXXXXXKXXXXXXXFXXLXLXXGXXXVHGXGXXXDXX------
yeast Emp46     MTTRKTASSLQLLGKITGTKAGTKQKKMNFINGLIWLYMCVWMVHGKVTQKDEL------ 54
yeast Emp47     ----------------MMMLITMKSTVLLSVFTVLATWAGLLEAHPLGDTSDAS------ 38
human ERGIC-53   ---------------MAGSRQRGLRARVRPLFCALLLSLGRF-VRGDGVGGDPAVALPHR 44

Consensus       KXXXXYSL--PXLX---XXXKXXXXWXXGXXAXLEEGRIVLTPX-KNXKGSLWLKXXXXX
yeast Emp46     KWNKGYSL--PNLLEVTDQQKELSQWTLGDKVKLEEGRFVLTPG-KNTKGSLWLKPEYSI 111
yeast Emp47     KLSSDYSL--PDLI---NARKVPNNWQTGEQASLEEGRIVLTSK-QNSKGSLWLKQGFDL 92
human ERGIC-53   RFEYKYSFKGPHLV---QSDGTVPFWAHAGNAIPSSDQIRVAPSLKSQRGSVWTKTKAAF 101

Consensus       KDXXTXEWTFRSXGXXGXTXGGLAFWXXQ-XNXGXXXXLFGGXX-XXNGLXILXDXXDXL
yeast Emp46     KDAMTIEWTFRSFGFRGSTKGGLAFWLKQ-GNEGDSTELFGGSSKKFNGLMILLRLDDKL 170
yeast Emp47     KDSFTMEWTFRSVGYSGQTDGGISFWFVQDSNVPRDKQLYNGPV-NYDGLQLLVDNNGPL 151
human ERGIC-53   EN-WEVEVTFRVTGRGRIGADGLAIWYAE--NQGLEGPVFGSAD-LWNGVGIFFDSFDND 157

Consensus       G----PXXXXXLNDGQKXXDXXXXXYXQXFASCLXXYQDSXVPSTXRXTYX------XXD
yeast Emp46     G----ESVTAYLNDGTKDLDIESSPY---FASCLFQYQDSMVPSTLRLTYN------PLD 217
yeast Emp47     G----PTLRGQLNDGQKPVD-KTKIYDQSFASCLMGYQDSSVPSTIRVTYD------LED 200
human ERGIC-53   GKKNNPAIVIIGNNGQIHYDHQNDGASQALASCQRDFRNKPYPVRAKITYYQNTLTVMIN 217

Consensus       NXLLKXQXDNXVCFQTRKVXFPXSGXFRIGXSAXNGAXXXXXESFEILKMQLXX-GVIED
yeast Emp46     NHLLKLQMDNRVCFQTRKVKFMGSSPFRIGTSAINDA---SKESFEILKMKLYD-GVIED 273
yeast Emp47     DNLLKVQVDNKVCFQTRKVRFP-SGSYRIGVTAQNGAVNNNAESFEIFKMQFFN-GVIED 258
human ERGIC-53   NGFTPDKNDYEFCAKVENMIIPAQGHF--GISAATGGLADDHDVLSFLTFQLTEPGKEPP 275

Consensus       SLIPNVNXMGQPKXXTKXIXXQTGXEKXXEKX----------PFXXXXESITXXEL---F
yeast Emp46     SLIPNVNPMGQPRVVTKVINSQTGEESFREKM----------PFSDKEESITSNEL---F 320
yeast Emp47     SLIPNVNAMGQPKLITKYIDQQTGKEKLIEKT----------AFDADKDKITNYEL---Y 305
human ERGIC-53   TPDKEISEKEKEKYQEEFEHFQQELDKKKEEFQKGHPDLQGQPAEEIFESVGDRELRQVF 335

Consensus       EKXNR--LEGKILANDIDXLL------------------------------XKLNXVVKX
yeast Emp46     EKMNK--LEGKIMANDIDPLL------------------------------RKMNKIVEN 348
yeast Emp47     KKLDR--VEGKILANDINALE------------------------------TKLNDVIKV 333
human ERGIC-53   EGQNRIHLEIKQLNRQLDMILDEQRRYVSSLTEEISKRGAGMPGQHGQITQQELDTVVKT 395

Consensus       QXELLXXXXXXXXXX--XPXXXLXKGXXXDDSXXXXXEXFQDFLSINXXLXXVXXE----
yeast Emp46     ERELIQRL---------RPLLDLKKTAISDDS-------FQDFLSMNANLDRLIKE---- 388
yeast Emp47     QQELLSFMTTITKQLSSKPPANNEKGTSTDDAIAEDKENFKDFLSINQKLEKVLVE---- 389
human ERGIC-53   QHEILRQVNEMKNSM--SETVRLVSGMQHPGSAGGVYETTQHFIDIKEHLHIVKRDIDNL 453

Consensus       -QEKXRXXXKXXGXXXPXXDEIXSKXXXWLXXLIFIMLXXAYYMFRIXQEIXKXKLL
yeast Emp46     -QEKIRQDAKLYGKQTKGHDEIFSKISVWLALLIFIMITLAYYMFRINQDIKKVKLL 444
yeast Emp47     -QEKYREATKRHGQDGPQVDEIARKLMIWLPLLIFIMLVMAYYTFRIRQEIIKTKLL 445
human ERGIC-53   VQRNMPSNEKPKCPELPPFPSCLSTVHFIIFVVVQTVLFIGYIMYRSQQEAAAKKFF 510
```
